# Hypouricemic Effect of Submerged Culture of *Ganoderma lucidum* in Potassium Oxonate-Induced Hyperuricemic Rats

**DOI:** 10.3390/metabo12060553

**Published:** 2022-06-16

**Authors:** Chung-Hsiung Huang, Tzu-Yu Chen, Guo-Jane Tsai

**Affiliations:** 1 Department of Food Science, National Taiwan Ocean University, Keelung 20224, Taiwan; huangch@mail.ntou.edu.tw (C.-H.H.); ashley8329@gmail.com (T.-Y.C.); 2 Center for Marine Bioscience and Biotechnology, National Taiwan Ocean University, Keelung 20224, Taiwan

**Keywords:** extracellular polysaccharides, hyperuricemia, *Ganoderma lucidum*, mycelium, submerged culture

## Abstract

Hyperuricemia is a disease caused by a high level of uric acid in the blood. It is an important factor for gout and may be linked to renal and hepatic failure. The objective of this study was to investigate the hypouricemic effects of submerged culture of *Ganoderma lucidum*. The lyophilized powder of mycelium (GM) and extracellular polysaccharides (GP) of the *G. lucidum* submerged culture were prepared. The contents of hypouricemic components, including phenolics and flavonoids, in GM (34.33 ± 0.41 mg/g and 0.32 ± 0.01 mg/g) were higher than that in GP (20.52 ± 1.49 mg/g and not detected). The hypouricemic effect of GM and GP was evaluated in potassium oxonate (PO)-injected rats. The average food intake (23.3 ± 1.2 g/day) and body weight (355.7 ± 28.0 g) were decreased, and the serum level of uric acid (5.56 ± 0.41 mg/dL) was increased in PO-injected rats. However, allopurinol (10 mg/kg b.w.) or GM treatment (200 or 400 mg/kg b.w) improved food intake (26.3 ± 2.7 g/day) and reduced the level of uric acid (4.45 ± 0.46 mg/dL). In parallel, the activity of hepatic xanthine oxidase (XOD) was downregulated from 841.29 ± 299.58 μU/mg protein to 540.80 ± 199.20 μU/mg protein. Moreover, GM and GP (200 or 400 mg/kg b.w) alleviated the level of blood urea nitrogen (BUN) from 30.49 ± 4.71 to 21.16 ± 4.25 mg/dL. GP treatment also diminished the level of alanine transaminase (ALT) from 52.63 ± 18.82 to 27.35 ±6.82 U/L. These results clearly demonstrated the hypouricemic effect of submerged *G. lucidum* culture and their potential against hyperuricemia-associated renal and hepatic damage. GM was more potent to alleviate hyperuricemia, and GP was more potent to improve renal and hepatic function.

## 1. Introduction

Hyperuricemia, a metabolic disease characterized by abnormally elevated uric acid levels in the blood, is present in 5–30% of the general population and seems to be increasing worldwide [1]. It has been reported that hyperuricemia increases the risk of gout, diabetes, renal, hepatic, and cardiovascular diseases [2]. Uric acid is the end product of purine degradation, and its homeostasis depends on the balance between production and elimination. A previous study showed that decreased urate clearances were found in mostly hyperuricemic patients, indicating the important role of renal urate handling [3].

Xanthine oxidase (XOD) is a key enzyme acting at the end of the purine nucleotide catabolism. The major function of hepatic XOD is to catalyze the oxidation of hypoxanthine and xanthine to uric acid [4]. XOD inhibitors could block the biosynthesis of uric acid from purine derivatives. Therefore, the development of XOD inhibitors is considered as one of the treatment methods for hyperuricemia [5]. Potassium oxonate (PO), a selectively competitive uricase inhibitor, suppresses the effect of hepatic uricase and produces hyperuricemia in rodents [6]. PO-induced hyperuricemia in rodents serves as a useful animal model to evaluate the efficacy of therapeutic agents against hyperuricemia and associated complications. So far, the strategies for hyperuricemia have focused on the use of XOD inhibitors, such as allopurinol, to reduce the uric acid production and the use of uricosuric agents to promote uric acid excretion [7]. However, 25–50% of hyperuricemic patients show poor response or intolerance to the recommended dose of the urate-lowering drug [8]. In addition, allopurinol has potential to elicit gastrointestinal symptoms, rash, chronic kidney disease, and hypersensitivity syndrome [8]. Therefore, development of more effective and less toxic hypouricemic agents and functional foods is urgently needed. 

Traditional Chinese medicine, including *Ganoderma lucidum*, is frequently utilized for the prevention of chronic disorders, such as chronic hepatitis and nephritis [9,10,11,12,13]. *G. lucidum* is an edible basidiomycete and saprophytic fungus and wildly used in oriental medicine because of its numerous pharmacological effects, e.g., antitumor, antiviral, immunomodulatory, and antihypertension activities [14]. To date, more than 400 secondary metabolites have been isolated from *G. lucidum*, and polysaccharides, triterpenoids, phenols, flavonoids, and peptides are the major bioactive metabolites contributing to the pharmacological effects [14,15]. The *Ganoderma* is made up of a fruiting body (the basidiocarp), a mycelium, and spores. In contrast to the cultivation of fruiting bodies and spores, submerged culture has the advantages of lower production and time costs, less space requirement, easier to control culture conditions, and higher yields, purity, and rejuvenative capacity [16]. Recently, our group has reported the hypoglycemic and antioxidant effects of submerged *G. lucidum* culture in type 2 diabetic rats [17]. Moreover, administration of submerged *G. lucidum* culture to diabetic rats also exhibited kidney- and liver-protective effects [17]. Notably, kidney and liver are the major organs affecting hyperuricemia. Therefore, we hypothesized that *G. lucidum* might be effective for hyperuricemia since it was recorded as a diuresis agent in Chinese herbal classic literature and closely associated with the prevention of hyperuricemia [18].

In this study, we prepared submerged culture of *G. lucidum* mycelium (GM) and extracellular polysaccharides (GP), followed by detecting the contents of phenolics and flavonoids. The hypouricemic effects of GM and GP were examined in PO-induced hyperuricemic rats via evaluating the serum level of uric acid and hepatic activity of XOD. In addition, the levels of serum urea nitrogen (BUN), creatinine, alanine aminotransferase, (ALT) and aspartate aminotransferase (AST) were assayed, and the weight of kidney and liver was measured to understand the protective effects of submerged *G. lucidum* culture against hyperuricemia-associated renal and hepatic damage.

## 2. Results

### 2.1. Mycelia (GM) and Polysaccharide (GP) Levels in the Submerged G. lucidum Culture and the Contents of Total Phenolic and Flavonoids

After incubation of submerged G. lucidum culture for 7 days, the whole culture, GM and GP were harvested and lyophilized. The levels of GM and GP contained in the submerged G. lucidum culture were determined as 3.84 ± 0.37 and 0.21 ± 0.04 g/L, respectively (Table 1). The contents of total phenols in whole culture, GM, and GP samples were 24.57 ± 0.71, 34.33 ± 0.41, and 20.52 ± 1.49 mg GAE/g dry weight (Table 1). The content of flavonoids in GM samples was 0.32 ± 0.01 mg QE/g dry weight, but the content of flavonoids in the whole culture and GP samples was too low to be detected (Table 1). These results show that the GM samples contained the highest phenolic and flavonoid content.

### 2.2. Influence of GM and GP on Daily Food Intake, Drinking Water Intake, and Body Weight of Hyperuricemic Rats

**Figure 1:**
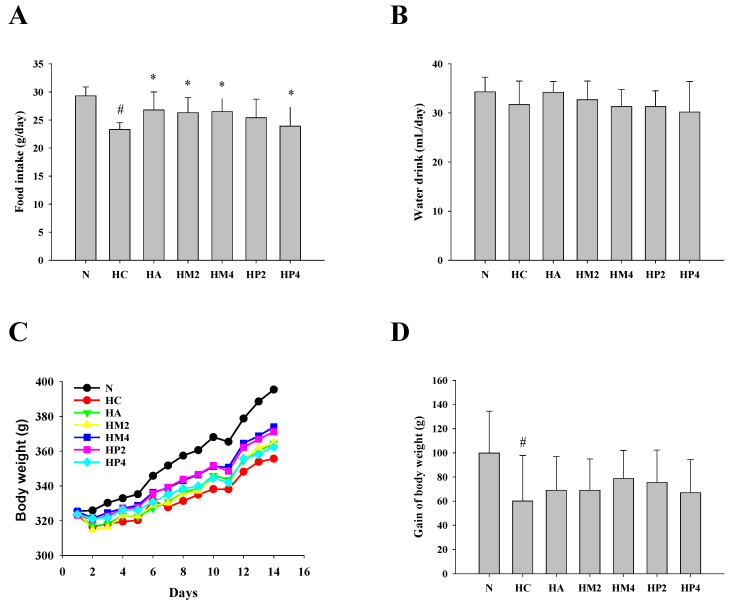
The average daily food intake, drinking water intake, and body weight of hyperuricemic rats. The rats were injected with PO and treated with allopurinol (HA; 10 mg/kg b.w.), GM (HM2 and HM4; 200 and 400 mg/kg b.w.), or GP (HP2 and HP4; 200 and 400 mg/kg b.w.), and the (**A**) average daily food intake, (**B**) drinking water intake, (**C**) body weight, and (**D**) gain of body weight were determined as described in the Materials and Methods. Results are expressed as mean ± S.D. for each group of rats (*n* = 8). # *p* < 0.05 compared with normal control (N). * *p* < 0.05 compared with hyperuricemic control (HC).

Compared to that of the normal control rats, daily food intake of PO-induced hyperuricemic control rats (HC group) was significantly decreased. However, the hyperuricemic rats treated with allopurinol (HA), GM (HM), or GP (HP) showed higher food intake than rats in the HC group (Figure 1A). The level of drinking water intake was comparable between each group (Figure 1B). Notably, gain of body weight was markedly decreased in the HC group. However, allopurinol, GM, or GP treatment could improve PO-induced decrement in body weight (Figure 1C,D).

### 2.3. Effects of GM and GP on Modulating Serum Level of Uric Acid and Hepatic Activity of XOD

**Figure 2:**
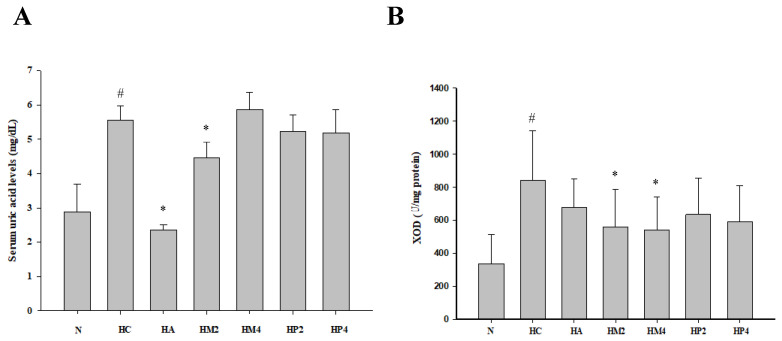
The level of serum uric acid and activity of hepatic xanthine oxidase (XOD) of hyperuricemic rats. The rats were injected with PO and treated with allopurinol (HA; 10 mg/kg b.w.), GM (HM2 and HM4; 200 and 400 mg/kg b.w.), or GP (HP2 and HP4; 200 and 400 mg/kg b.w.), and the level of (**A**) serum uric acid and activity of (**B**) hepatic XOD were determined as described in the Materials and Methods. Results are expressed as mean ± S.D. for each group of rats (*n* = 8). # *p* < 0.05 compared with normal control (N). * *p* < 0.05 compared with hyperuricemic control (HC).

As shown in Figure 2, PO injection significantly elevated serum level of uric acid and hepatic activity of XOD compared to normal control. However, either allopurinol or GM treatment could alleviate the level of uric acid, indicating the hypouricemic effect of GM (Figure 2A). Interestingly, only GM, but rather than allopurinol, significantly diminished XOD activity (Figure 2B), revealing that the action mechanism of allopurinol and GM may be distinct.

### 2.4. Protective Effects of GM and GP against Hyperuricemia-Associated Kidney and Liver Injury

**Figure 3:**
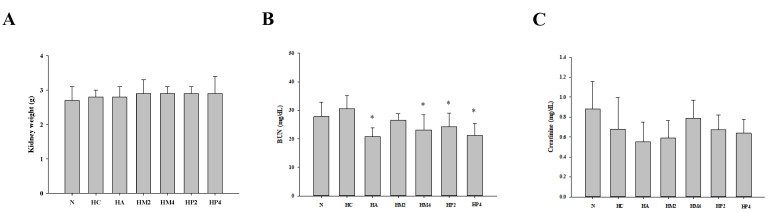
The weight of kidney and levels of blood urea nitrogen (BUN) and creatinine of hyperuricemic rats treated with G. lucidum formula. The rats were injected with PO and treated with G. lucidum formula, and the (**A**) weight of kidney and serum level of (**B**) BUN and (**C**) creatinine were determined as described in the Materials and Methods. Results are expressed as mean ± S.D. for each group of rats. * *p* < 0.05 compared with hyperuricemic control (HC).

**Figure 4:**
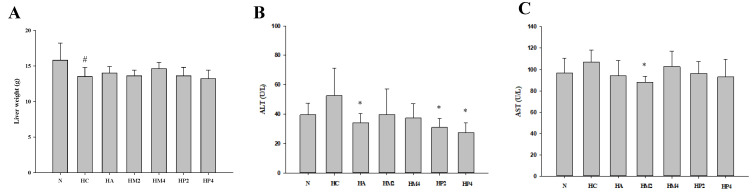
The weight of liver and levels of alanine aminotransferase (ALT) and aspartate aminotransferase (AST) of hyperuricemic rats treated with G. lucidum formula. The rats were injected with PO and treated with G. lucidum formula, and the (**A**) weight of liver and serum level of (**B**) AST and (**C**) ALT were determined as described in the Materials and Methods. Results are expressed as mean ± S.D. for each group of rats. # *p* < 0.05 compared with normal control (N). * *p* < 0.05 compared with hyperuricemic control (HC).

Although PO injection and submerged culture of G. lucidum formula treatment had no significant influence on the kidney weight (Figure 3A), the level of serum BUN of rats in HA, HM4, HP2, and HP4 groups was statistically lower compared to that in the HC group, revealing the kidney-protective potential of GM and GP (Figure 3B). A similar trend could be observed in the analysis of serum creatinine, in spite of the fact that there was no significant difference between each group (Figure 3C). With respect to the liver, the weight of liver was markedly decreased, and the levels of ALT and AST were higher in rats of the HC group compared to that of the normal control (Figure 4). Although both allopurinol and *G. lucidum* formula had no obvious impact on ameliorating the decrement in liver weight, diminished levels of ALT and AST were observed in the rats of HM and HP groups, revealing the liver-protective potential of GM and GP (Figure 4). 

## 3. Discussion

In this study, we investigated the hypouricemic activities of submerged *G. lucidum* culture in a rat model of PO-induced hyperuricemia. PO-induced hyperuricemia was further confirmed by a dramatic increase in the serum level of uric acid and hepatic activity of XOD. The hypouricemic effect of submerged *G. lucidum* culture was evidenced by a remarkably lower level of uric acid and XOD in rats treated with GM and GP, and the potential mechanism was correlated to the regulation of key enzyme XOD.

Hyperuricemia is considered an important factor linked to renal and hepatic failure [2]. Moreover, PO-injection not only induced hyperuricemia but also elicited oxidative stress and related organ damage [19]. As the antioxidant activity of submerged *G. lucidum* culture has been substantiated [17], renal and hepatic function of PO-injected rats were evaluated by serum biochemistry tests. Creatinine and BUN are critical indicators for renal function, as its impairment is usually accompanied by an increase in the level of the two indicators in serum. Due to the lower dosage of PO, the impairment of kidney by PO in hyperuricemia rats was not obvious in this model. However, administration of GM and GP diminished the level of BUN, indicating a protective effect of submerged *G. lucidum* culture against the development of hyperuricemia-associated renal damage [20]. Improvement in renal function is also helpful for uric acid excretion. On the other hand, the serum level of uric acid is associated with the development of cirrhosis and the presence of elevated serum level of liver enzymes [21]. In the current study, the value of AST was statistically downregulated only in the HM2 group, whereas the level of ALT significantly declined in rats of HP2 and HP4 groups. It has been well known that ALT is primarily localized to the liver but the AST is present in a wide variety of tissues [22]. Accordingly, we suggest the data of ALT is more valuable to evaluate hepatic function compared to that of AST. Moreover, oxidative stress elicited by the employed animal model may not be violent enough to damage the mitochondria, since ALT and AST are critical enzymes prevalently located in the hepatocyte cytoplasm and mitochondria, respectively [22]. 

Until now, limited information pertaining to the hypouricemic effects of *Ganoderma* species is available. In 2018, Yong et al. have reported the hypouricemic effects of *G. applanatum* in hyperuricemic mice. Both ethanol and water extracts (30–120 mg/kg) could decrease serum level of uric acid [10]. However, the extracts could not suppress XOD activity. Alternatively, hypouricemic effects of *G. applanatum* were majorly mediated by modulating renal organic anion transporter 1, glucose transporter 9, uric acid transporter 1, and gastrointestinal concentrative nucleoside transporter 2 that might promote urine uric secretions and hinder purine absorption in the alimentary canal [10]. More recently, the same group reported the hypouricemic activity of 2,5-dihydroxyacetophenone (DHAP), a compound derived from *G. applanatum*. DHAP administration (20, 40, and 80 mg/kg) reduced serum level of uric acid, BUN, and creatinine in hyperuricemic mice. XOD inhibition was suggested as a possible action mechanism for the hypouricemic effect of DHAP because of the downregulated activity of XOD [9].

Polysaccharides have been demonstrated as one of the bioactive components contributing to the hypouricemic effect of medicinal fungi. For example, exopolysaccharides derived from *Cordyceps militaris* (400 mg/kg) showed a similar effect with allopurinol (5 mg/kg) on modulating serum levels of uric acid, BUN, and hepatic XOD activities in hyperuricemic mice [11]. In addition to polysaccharides, numerous studies have shown that flavonoids in functional foods have hypouricemic activity by reducing uric acid synthesis, increasing renal uric acid secretion, and preventing renal reabsorption of uric acid [19,23,24]. Moreover, phenolic substances derived from natural products, such as adlay bran, *Mesona procumbens* Hemsl., and *Fraxinus angustifolia*, also exhibited significant uric acid suppression and XOD inhibition activities [25,26,27]. In this study, GM showed a higher potency to alleviate the levels of uric acid and XOD, and GP more evidently downregulated the levels of BUN and ALT. Since flavonoids were only detectable in GM, we suggest that flavonoids may be the major bioactive components responsible for the hypouricemic activity, and polysaccharides and phenolics are primary components contributing to kidney- and liver-protective effects of submerged *G. lucidum* culture. However, the potency and synergistic effect of these metabolites, including flavonoids, polysaccharides, and phenolics, on improving hyperuricemia warrants further investigation. In addition to XOD activity, further efforts are needed to investigate the impact of these active ingredients on renal uric acid secretion and reabsorption for a comprehensive understanding of the action mechanism of submerged *G. lucidum* culture.

## 4. Materials and Methods

### 4.1. Culture, Chemicals, and Reagents

*G. lucidum* BCRC 36123 was obtained from Bioresources Collection and Research Center (Hsinchu, Taiwan). All chemicals and reagents were purchased from Sigma-Aldrich Chemical Co. (St. Louis, MO, USA) unless otherwise stated. Standard chow diet was purchased from PMI Nutrition International, LLC (Arden Hills, MN, USA).

### 4.2. Submerged Culture of G. lucidum mycelia (GM) and Extracellular Polysaccharides (GP) Samples Preparation

Based on the method described by Huang et al. [17], two mycelial pieces (1 × 1 cm^2^) *G. lucidum* mycelium pad grown on Gano medium agar plates were added into a flask containing 250 mL Gano medium and incubated at 30 °C, 110 rpm for 7 days. Based on the method described previously, the *Ganoderma* mycelia and extracellular polysaccharides in culture were separated and measured [28]. Briefly, *G. lucidum* culture was centrifuged (4000× *g*, 20 min) to collect mycelium and supernatant, respectively. The collected mycelium was washed with deionized water twice and freeze-dried (designated as GM). The supernatant was supplemented with 95% ethanol (1:4, *v*/*v*) to precipitate extracellular polysaccharides. After being washed with 75% ethanol twice, the polysaccharides were freeze-dried (designated as GP). In addition, the *G. lucidum* whole culture was directly freeze-dried. The freeze-dried samples of whole culture, GM, and GP were stored at −20 °C for experiments.

### 4.3. Determination of Total Phenolic Contents

Total soluble phenolics in whole culture, GM, and GP were determined with Folin–Ciocalteu reagent, and gallic acid was utilized as a standard phenolic compound as described in the previous study [29]. Briefly, 1 mg of lyophilized samples was mixed with distilled water (45 mL) and added with 1 mL of Folin–Ciocalteu reagent. After mixing thoroughly for 3 min, 3 mL of Na_2_CO_3_ (2%) was added. After standing for 2 h with intermittent shaking in dark, the optical density was measured at 760 nm. The concentration of total phenolic contents was determined as milligrams of gallic acid equivalent (GAE) based on the calibration curve of gallic acid standards [30].

### 4.4. Determination of Flavonoid Contents

Lyophilized GM and GP samples were dissolved in distilled water, and the diluted solution of GM, GP, or quercetin (0.5 mL) were added with 95% ethanol (1.5 mL), 10% aluminum nitrate (0.1 mL), 1 M potassium acetate (0.1 mL), and distilled water (2.8 mL). After incubation for 40 min at room temperature, the optical density was measured at 415 nm. The concentration of flavonoid contents was determined as milligrams of quercetin equivalent (QE) based on the calibration curve of quercetin standards.

### 4.5. Animals and Experimental Design

Conventional male Sprague Dawley rats (7 weeks old) were purchased from BioLASCO Taiwan Co., Ltd. (Taipei, Taiwan). The rats were housed in stainless steel cages and fed ad libitum in a climate-controlled warm animal room. All animal experiments were approved by the Institutional Animal Care and Use Committee of the National Taiwan Ocean University (NTOU-106017), and the rats were handled in accordance with the guidelines of the Declaration of Helsinki.

**Figure 5:**
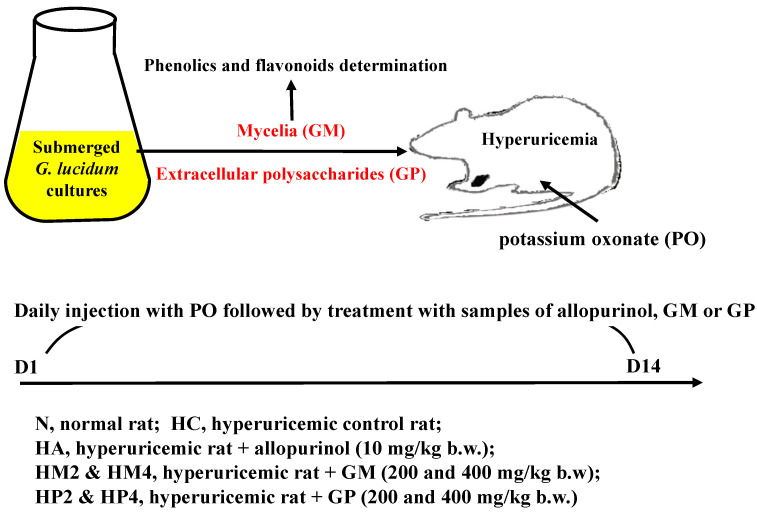
Protocol of hyperuricemia induction and treatment of rats. Sprague-Dawley rats were intraperitoneal injected with potassium oxonate (PO; 250 mg/kg b.w.) daily to induce hyperuricemia. The rats in the normal (N) group did not receive any treatment throughout the animal experiment. PO-injected rats were randomly divided to 6 groups (*n* = 8): hyperuricemic control (HC), daily treated with allopurinol (10 mg/kg b.w.; HA), daily treated with GM (200 or 400 mg/kg b.w.; HM2 or HM4), or daily treated with GP (200 or 400 mg/kg b.w.; HP2 or HP4). Except for the rats in N and HC groups, the other rats were orally treated with allopurinol or G. lucidum formula daily. The rats were treated for 2 weeks and then sacrificed on day 15 to individually isolate and weigh the kidney and liver. The blood samples, as well as liver samples, were harvested for further analysis.

After adaptation for 1 week, except for normal control rats, the other rats were intraperitoneally injected with uricase inhibitor potassium oxonate (PO; 250 mg/kg b.w.) daily to induce hyperuricemia [31] As shown in Figure 5, the rats in the normal group (N) did not receive any treatment throughout the animal experiment. PO-injected rats were randomly divided to 6 groups (*n* = 8): hyperuricemic control without any treatment (HC), daily treated with allopurinol (10 mg/kg b.w.; HA), daily treated with GM (200 or 400 mg/kg b.w.; HM2 or HM4), or daily treated with GP (200 or 400 mg/kg b.w.; HP2 or HP4). Except for the rats in N and HC groups, the other rats were orally treated with allopurinol GM or GP 1 h after PO-injection. The rats were treated for 2 weeks and then sacrificed on day 15 to individually isolate and weigh the kidney and liver. The blood samples, as well as liver samples, were harvested for further analysis.

### 4.6. Evaluation of the Serum Levels of Uric Acid, BUN, CREA, AST, ALT, and Hepatic Level of XOD

Serum levels of uric acid, BUN, creatinine, AST, ALT, and XOD activity of liver homogenates were measured using a uric acid kit, Enzymatic Kits (Randox Laboratories Limited, Crumlin, UK) and a XOD kit (Cayman Chemical, Ann Arbor, MI, USA), respectively, according to the manufacturer’s instructions.

### 4.7. Statistical Analysis

Data were analyzed statistically using SPSS Version 12.0 (SPSS Inc., Chicago, IL, USA). One-way analysis of variance (ANOVA) was used to determine statistical differences between sample means, with the level of significance set at *p* < 0.05. Multiple comparisons of means were undertaken using Duncan’s multiple range test. All data are expressed as mean ± SD.

## 5. Conclusions

Our findings provide the first evidence to demonstrate the improvement in hyperuricemia and renal and hepatic function in PO-injected rats treated with submerged *G. lucidum* culture. Downregulation of XOD activity is suggested as one of the mechanisms for its hypouricemic activity. Since submerged cultures have numerous advantages over basidiocarp cultivation, submerged *G. lucidum* culture-derived mycelium and extracellular polysaccharides have potential to be developed as functional foods for controlling hyperuricemia and preventing further complications.

## Figures and Tables

**Table 1:** Contents of phenolics and flavonoids in the lyophilized powder of submerged culture of *Ganoderma lucidum,* mycelium, and extracellular polysaccharides.

Item	Mycelium (g/L)	Extracellular Polysaccharides (g/L)
*Ganoderma lucidum*	3.84 ± 0.37	0.21 ± 0.04
**Sample**	**mg/g dry weight**	
	**Phenolics (gallic acid equivalent)**	**Flavonoids (quercetin equivalent)**
Whole culture	24.57 ± 0.71 ^a^	N.D.
Mycelium	34.33 ± 0.41 ^b^	0.32 ± 0.01
Extracellular polysaccharides	20.52 ± 1.49 ^c^	N.D.

Results are expressed as mean ± S.D. (*n* = 3). Different letters a–c mean significant difference (*p* < 0.05). N.D.: not detected.

## Data Availability

Data for this study are available upon request from the corresponding author.
